# Successful endovascular repair of iliac artery aneurysms with unsuitable anatomy by combining unibody bifurcated endograft and iliac branch systems to preserve hypogastric artery blood flow: a report of two cases

**DOI:** 10.1186/s13019-022-01855-1

**Published:** 2022-05-03

**Authors:** Daisuke Akagi, Kai Murase

**Affiliations:** 1 Department of Vascular Surgery, Tokyo Metropolitan Geriatric Medical Center, Tokyo, Japan; 2 Department of Cardiovascular Surgery, Kawasaki Medical School, 577 Matsushima, Kurashiki-city, Okayama Japan; 3 Department of Surgery, Tokyo Metropolitan Geriatric Medical Center, Tokyo, Japan

**Keywords:** Endovascular repair, Iliac artery aneurysm, Hypogastric artery, Customized endograft therapy, Case reports

## Abstract

**Background:**

To overcome the anatomical limitation of a narrow aorta and short length from the renal artery to the terminal aorta, unibody endograft AFX2 and iliac branch endoprosthesis (IBE) can be combined.

**Case presentation:**

Case 1: The first patient was an 89-year-old woman who had a right saccular common iliac artery (CIA) aneurysm (38 mm); the abdominal aorta was not aneurysmal (diameter, 19 mm). The right CIA’s origin was 10 mm in diameter. A bifurcated AFX2 was placed in an ordinary manner. Then, IBE was inserted in the right leg of the AFX2. Case 2: The second patient was an 87-year-old man diagnosed with an abdominal aortic aneurysm (55 mm), right dissecting CIA aneurysm (20 mm), and right hypogastric artery aneurysm (22 mm) extending to the bifurcation of the superior and inferior gluteal arteries. The length between the renal artery and terminal aorta was 107 mm. The beginning of the right CIA was segmentally stenotic (13 mm). A bifurcated AFX2 was placed in the infrarenal aorta; IBE was advanced to the origin of the right limb of the AFX2. To control the type 1b endoleak, the right superior gluteal artery was embolized with coils and internal iliac components were deployed toward the inferior gluteal artery. Satisfactory results were obtained in both cases.

**Conclusion:**

The AFX2 main body and IBE could be combined to preserve hypogastric blood flow and overcome anatomical limitations.

## Introduction

Endovascular iliac arterial aneurysm repair has advantages over open repair with regard to surgical invasiveness [1–4]; hypogastric artery embolization is a commonly-adopted technique for this procedure [2]. Sacrificing hypogastric arteries results in ischemic events, such as colitis and buttock claudication [3–9], which adversely impact quality of life. To maintain hypogastric artery blood flow, the iliac branch device (IBD) is an effective and viable option in anatomically suitable cases [10–17]. In Japan, for the endovascular reconstruction of the hypogastric artery, only the iliac branch endoprosthesis (IBE) device (Gore, Flagstaff, AZ, USA) is available. There are many restrictions placed by the manufacturer on the use of IBE with the Excluder main body [13, 14], and appropriate preoperative planning in endovascular repair may yield satisfactory results. AFX2 (Endologix, Irvine, CA, USA) is a unique bifurcated unibody endograft that fits the small terminal aorta and provides a proper landing zone for the common iliac artery (CIA) [18, 19]. We report two cases of successful endovascular repair of iliac arterial aneurysms aiming to prevent pelvic ischemia. The patients provided consent for the publication of the case details and images.

## Case presentation

### Case 1

**Fig. 1:**
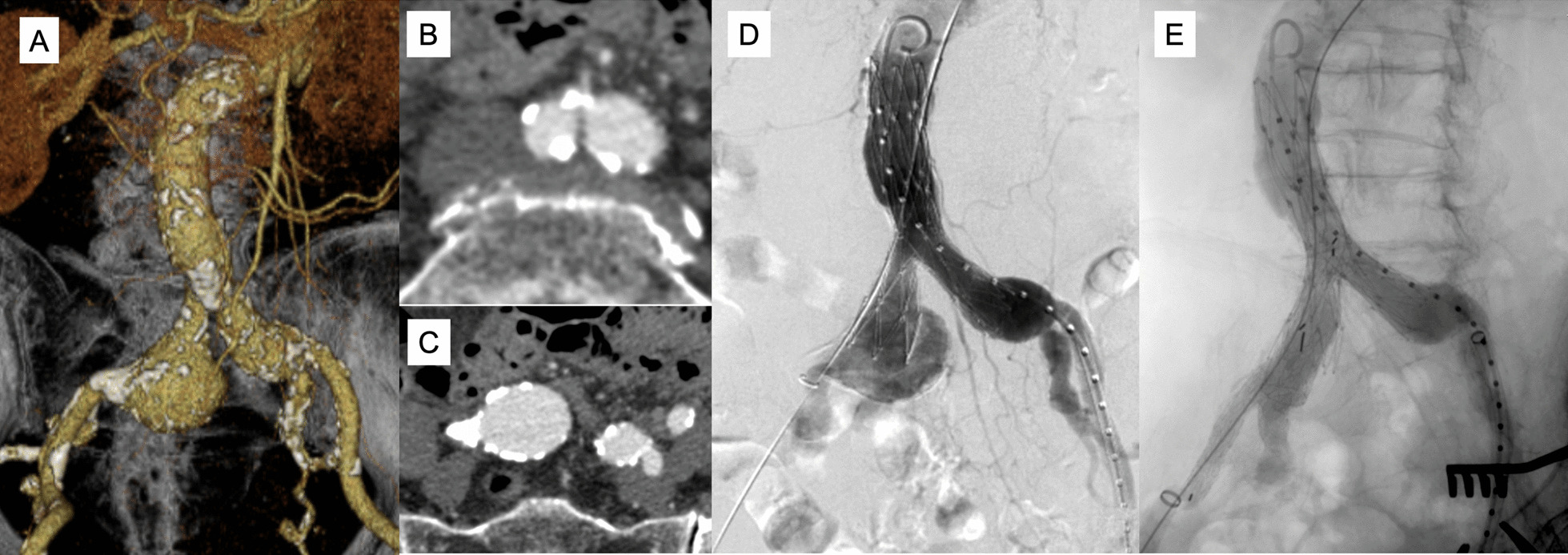
Case 1: **A**–**C** Preoperative computed tomography (CT) imaging. **A** CT angiography (CTA) imaging. **B** Aorta was not dilated and terminal aorta was narrow. **C** The proximal portion of right common iliac artery was narrow, measuring 10 mm. **D** The right common iliac artery aneurysm measuring 38 mm. **E**–**F** Intraoperative angiogram. **E** Aortogram after placement of AFX 2 bifurcated main body. **F** Completion angiogram showing successful deployment of AFX2 and Iliac branch endoporesis (IBE) and no endoleaks

An 89-year-old woman was referred to our department for treatment of a right saccular CIA aneurysm (38 mm). She was active despite her old age. The abdominal aorta was not aneurysmal; the terminal aorta was 19 mm in diameter. The origin of the right CIA had a diameter of 10 mm, and the length of the right CIA was 55 mm. The left CIA was only 18 mm in diameter (Fig. 1A–C). For endovascular treatment, as there was no proper landing zone at the proximal right CIA and the terminal aorta was normal and too narrow for a repair using the Excluder, we planned to use the AFX2 aorto-bi-iliac device and IBE landing in the right limb of the AFX2 to exclude the CIA aneurysm and preserve right hypogastric artery blood flow.

The AFX2 was deployed first and the IBE was placed subsequently (Fig. 1D, E). A 25 × 60 × 20 × 40-mm bifurcated AFX2 was loaded into the infrarenal aorta in the usual manner from the right common femoral artery (CFA). It was pulled down to aortic bifurcation and deployed. Next, a 23 × 12-mm IBE was deployed from the origin of the right limb of the AFX2 placed in advance. The curled pig-tail catheter was passed through the endoskeleton safely. The guidewire from the right CFA was safely caught above the bifurcated AFX2 with a snare catheter from the left CFA and tugged out. The IBE was advanced from the right CFA. Using the pull-through guidewire, the 12F sheath from the left CFA was advanced through the IBE. A directional catheter was used to cannulate the hypogastric artery. Using an Amplatz Super Stiff wire (Cook Medical, Bloomington, IN, USA), the two Gore internal iliac components were deployed (Fig. 1). Completion angiography demonstrated excellent filling of the aortic endograft, both iliac limbs, and right internal and external iliac arteries without opacification of the right CIA aneurysm and endoleaks. Follow-up computed tomography (CT) angiography findings revealed continued perfusion of the right hypogastric artery and bilateral external iliac arteries at 44 months postoperatively.

### Case 2

**Fig. 2:**
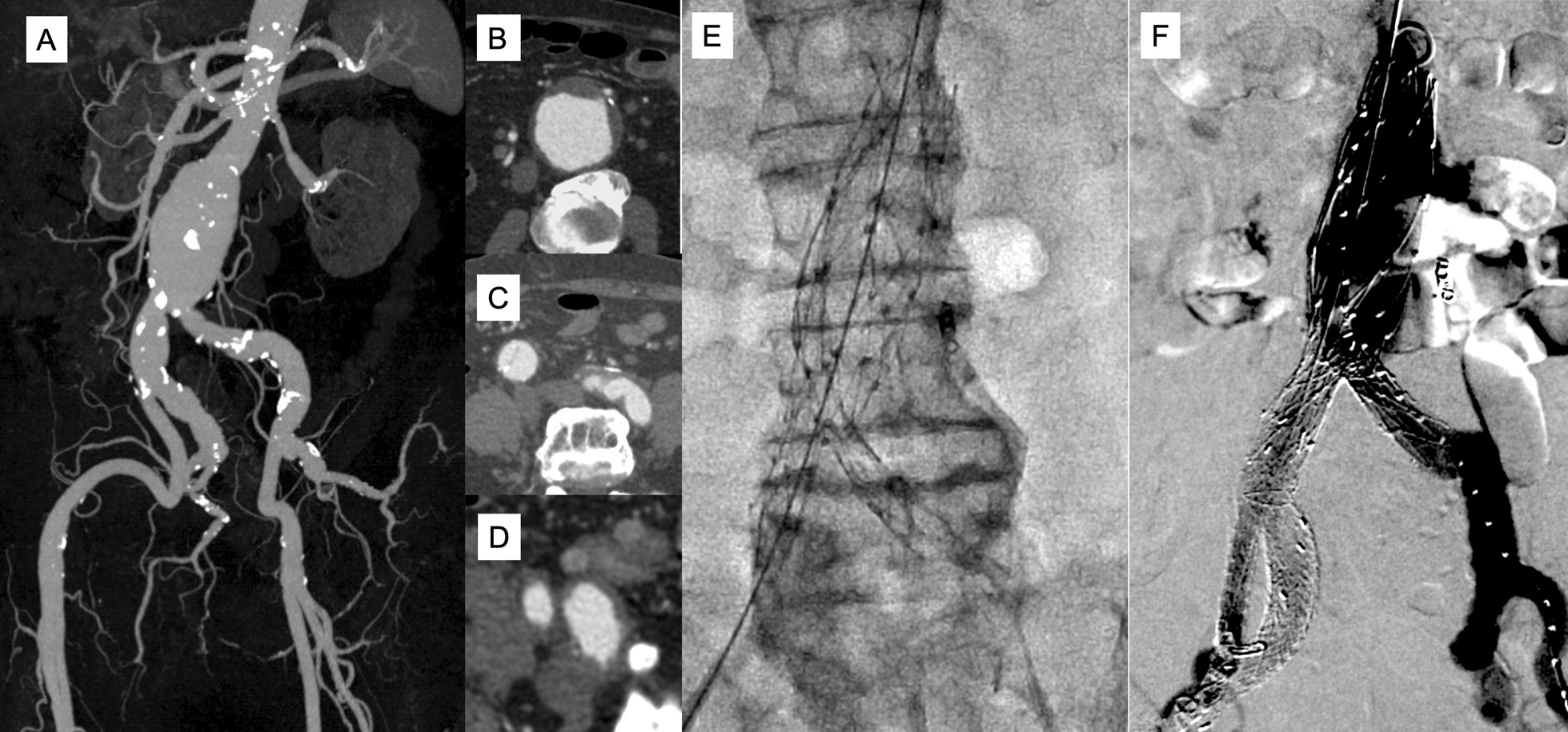
Case 2: **A**–**D** Preoperative computed tomography (CT) imaging. **A** CT angiography (CTA) imaging. The beginning of the right CIA was segmentally stenotic, measuring 13 mm in diameter. **B** Abdominal aortic aneurysm (55 mm). **C** The right common iliac artery showing dissecting aneurysm (20 mm). **D** The proximal portion of right hypogastric artery aneurysm (22 mm). **E**–**F** Intraoperative angiogram. **E** Fluorescent image after placement of AFX2 bifurcated main body. **F** Completion angiogram showing proper blood flow to right external and inferior gluteal artery without any endoleaks

The patient was an 87-year-old man with a history of coronary artery disease (treated via catheter intervention), chronic renal failure, and idiopathic thrombocytopenic purpura. He was diagnosed with an abdominal aortic aneurysm (55 mm), right dissecting CIA aneurysm (20 mm), and right hypogastric artery aneurysm (22 mm) extending to the bifurcation of the superior and inferior gluteal arteries (Fig. 2A–D). The infrarenal aorta was almost straight, although the distance between the lower renal artery and the terminal aorta was only 107 mm. The length of the right CIA was 56 mm, although the beginning of the right CIA was segmentally (length < 10 mm) stenotic, measuring 13 mm in diameter. The lengths of the infrarenal aorta and right CIA were shorter than was allowed for the use of the Excluder and IBE (165 mm). We planned to use the AFX2 aorto-bi-iliac device and IBE to exclude the aneurysms and to preserve direct flow to pelvis under embolization of the superior gluteal artery for obtaining sufficient sealing length of the distal internal iliac component.

A 28 × 100 × 20 × 40-mm bifurcated AFX2 was placed in the infrarenal aorta, with the limbs of the device extending into both CIAs (Fig. 2E). The IBE was advanced to the origin of the right limb of the AFX2. The right superior gluteal artery was embolized with proper coils to secure a proper distal landing zone and control a type 1b endoleak from the internal iliac components. Then, three Gore internal iliac components were deployed toward the inferior gluteal artery. Completion angiography demonstrated excellent results, with the total exclusion of the abdominal aortic and right iliac artery aneurysms with flow preserved in both, the right inferior gluteal and external iliac arteries (Fig. 2F). The patient was discharged without complications. He remained healthy, with no evidence of endoleaks or occlusion of the iliac branch at 24 months postoperatively.

## Discussion

Internal iliac artery embolization disrupts direct blood flow to pelvis and increases the risk of colonic ischemia, buttock claudication, erectile dysfunction, and spinal cord ischemia [5–8]. Buttock claudication due to bilateral hypogastric embolization occurs more frequently and is more severe than that caused by unilateral hypogastric embolization [9]. Schneider et al. reported excellent short- and long-term results with IBE to treat iliac artery aneurysms [11, 12]. Fargion et al. reported a high rate of freedom from occlusion of iliac branch device at 60 months (97.7%) [17]. However, there are many anatomical restrictions regarding IBE use [14, 15]. The distance from renal artery to iliac bifurcation is sometimes shorter than 165 mm, which is a contraindication as per the manufacturer’s instructions. To overcome this limitation, one option is to adopt the AFX2 main body combined with IBE. The aortic part of the AFX2 varies in length (60–100 mm) and can be used in patients with short aortas. The options for the iliac part of the AFX2 are 40 or 30 mm in length and 20 or 16 mm in diameter. The iliac part of the AFX2 has ideal dimensions for obtaining a long seal zone between the AFX2 and IBE that is 23 mm in diameter. To perform the repair with this combination, the IBE exoskeleton should be matched to the AFX2. The gap between the fabric and skeleton of the AFX2 may be a concern for type III endoleaks. This endoleak would be difficult to control because the skeletons disturb proper fitting of these two endografts. We did not observe any adverse outcomes because the narrow segment in the proximal CIA might tighten overlapping grafts. The range of proper proximal CIA diameter is unclear for this combination to work successfully. In our cases, the they were 10 mm and 13 mm. In diffusely large proximal CIA, combination of the AFX2 and IBE would not be indicated.

In small aorta, cannulation and construction of the contralateral limb are sometimes difficult for devices with two- or three-piece designs. Also, both limbs of endografts in such devices are compressed by the aortic walls and may cause lower limb ischemia. This combination of AFX2 and IBE is also indicated among patients with small caliber terminal aortas.

To provide a suitable proximal iliac landing zone, the customization of AFX2 and IBE was useful [20, 21]. The AFX2 main body and bilateral IBE can also be used [20]. Only these previous reports did not mention the necessity of the small caliber at proximal CIA [20, 21]. However, in all previous reports, the shapes of CIAs for IBE deployment were bell-bottoms with narrow beginnings. It may be helpful to consider the proper combination of the AFX2 and IBE for suitable cases. The thin-slice CT images should be carefully evaluated and measured to avoid performing this procedure in cases with anatomical risk.

There are some important technical notes to be considered for this procedure. First, the order of deployment is important. The AFX2 main body should be deployed first, prior to IBE, because the proximal size of IBE is 23 mm and the size of the AFX2 leg is 16 or 20 mm. Second, to prevent guidewires from getting caught in the endoskeleton when inserting a pull-through wire between the bilateral femoral arteries, a pig-tail catheter was passed in a curved shape and then, the wire was tagged above the AFX2 main body and pulled down.

## Conclusions

Knowing the characteristics of all devices, we can customize the use of various endovascular grafts for safe exclusion of the aneurysmal sac with preservation of the hypogastric artery to obtain good outcomes and minimize complications. Combining the AFX2 with IBE can provide stable repair of iliac artery aneurysms in the selected cases.

## Data Availability

All data in the study are included in this published article.

## Abbreviations

CIA: Common iliac artery
CFA: Common femoral artery
IAA: Iliac arterial aneurysm
IBD: Iliac branch device
IBE: Iliac branch endoprosthesis
